# From Innocuous to Aggressive: A Case of Odontogenic Keratocyst Transforming to Unicystic Ameloblastoma

**DOI:** 10.7759/cureus.78806

**Published:** 2025-02-10

**Authors:** Sharadindu M Kotrashetti, Shinju John, Vijayalakshmi Kotrashetti, Richa Mishra, Sakshi Panday

**Affiliations:** 1 Oral and Maxillofacial Surgery, KLE Vishwanath Katti Institute of Dental Sciences, Belagavi, IND; 2 Oral and Maxillofacial Pathology, Maratha Mandal's Nathajirao G. Halgekar Institute of Dental Sciences and Research Centre, Belagavi, IND

**Keywords:** cone beam computed tomography, cyst enucleation, mandible, odontogenic keratocyst, unicystic ameloblastoma

## Abstract

Odontogenic keratocysts are known for their aggressive and recurrent nature. Clinically and radiographically distinguishing between ameloblastoma and odontogenic keratocysts can be challenging due to their similar locations, age of affected patients, and multilocular appearance. Ameloblastomas co-occurring with odontogenic cysts or other odontogenic lesions have been documented as combined lesions. However, unique incidences of odontogenic keratocysts showing changes of unicystic ameloblastomas are scarce, especially a large aggressive lesion occurring in the mandible extending from the molar-to-molar tooth region in a young patient as presented in this case. This transformation is rare and intriguing and warrants detailed exploration and documentation. Considering the rarity of the lesion, this case highlights the importance of considering the aggressive nature of the lesion and its transformation into ameloblastoma, cautioning the surgeon to have a long-term follow-up to evaluate recurrence and emphasizing the regeneration and restoration of quality of life in young patients.

## Introduction

Odontogenic keratocyst (OKC) stands out among odontogenic cysts due to its distinct microscopic features, aggressive behavior, and high recurrence rate (6-62%) [1]. OKCs account for 7% to 11% of all oral developmental cysts. These cysts occur in the second and third decades of life with a slight male predominance and are typically found in the posterior mandibular region [2]. Most often, OKCs are asymptomatic and diagnosed on routine radiography. Radiographically, well-circumscribed unilocular/multilocular radiolucent lesions with smooth or loculated borders causing displacement or resorption of roots, without cortical bone expansion, are noted. Treatment modalities for OKCs can be conservative (enucleation with adjuvant therapy) or radical (peripheral osteotomy and marginal resection), depending on the size and extent of the lesion [3]. Brannon identified several factors contributing to the recurrence, including incomplete cyst removal, the friable nature of the capsule, bony perforation, adherence to surrounding tissues, and the presence of satellite cysts and type of epithelialization [4-6].

On review of the literature, only one case of OKC transforming into a plexiform type of luminal unicystic ameloblastoma was reported in a 35-year-old patient, located in the mandibular anterior region. In contrast, we report a unique case occurring in a 16-year-old patient with an extensive, aggressive lesion spanning from the molar-to-molar teeth region in the mandible, which was diagnosed as OKC transforming into unicystic ameloblastoma. Considering the age of the patient, enucleation was considered the treatment option.

## Case presentation

Clinical examination

**Figure 1:**
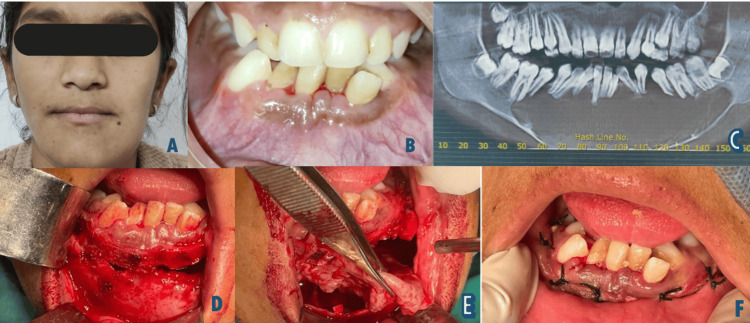
Pre-operative and intra-operative pictures of the patient A) Clinical picture showing the asymmetry of the face. B) Intra-oral picture showing the bony expansion noted in the mandibular anterior region. C) Orthopantomogram showing huge multilocular radiolucent lesion extending from 38-46. D) Intra-operative picture showing the labial plate exposure. E) Intra-operative picture showing the removal of cystic contents from the bony cavity. F) Intra-operative picture showing closure using black silk sutures after enucleation. Written informed consent to publish the patient's image in an open-source journal has been obtained from the patient's legal guardian.

A 16-year-old female presented to the outpatient Oral and Maxillofacial Surgery department, with swelling over her lower jaw region for two years. The swelling was asymptomatic and insidious in onset with a gradual increase in size. The patient had a normal build with no other significant medical history. Initial clinical examination revealed extraoral asymmetry measuring approximately 4 cm x 6 cm in the lower jaw region. Anterio-posteriorly, the swelling extended from the left mandibular body to the right mandibular body region (Figure 1A). Overlying skin appeared normal and non-adherent to deeper structures. On intra-oral examination, a diffuse enlargement was seen over the mandibular region. On palpation, the swelling was non-tender and bony hard in consistency, with a noticeable bony expansion and eggshell crackling phenomenon. The overlying mucosa appeared normal (Figure 1B). Cone beam computed tomography was advised, and the image showed a huge multilocular radiolucent lesion in the anterior and posterior mandible regions, displacing roots of the lower mandibular teeth extending from 38-46. It was suggestive of an expansile lesion with thinning and expansion of the cortical plates at labial and lingual cortices (Figure 1C). Based on clinical and radiographic findings, a differential diagnosis of a glandular odontogenic cyst, botryoid odontogenic cyst, OKC, ameloblastoma, and central giant cell granuloma was made. An incisional biopsy was performed, and it was histopathologically reported as an odontogenic cyst or tumor. The diagnosis was inclusive. Based on histopathology and radiographic and clinical investigations, treatment was planned.

Considering the age of this patient and the extent of the lesion, complete cyst enucleation of the lesion under general anesthesia was planned. Pediatric and pre-anesthetic clearance was obtained before the procedure. Parents were informed about the need for regular follow-up and the long-term healing process required for complete bone formation [7].

Surgical procedure

**Figure 2:**
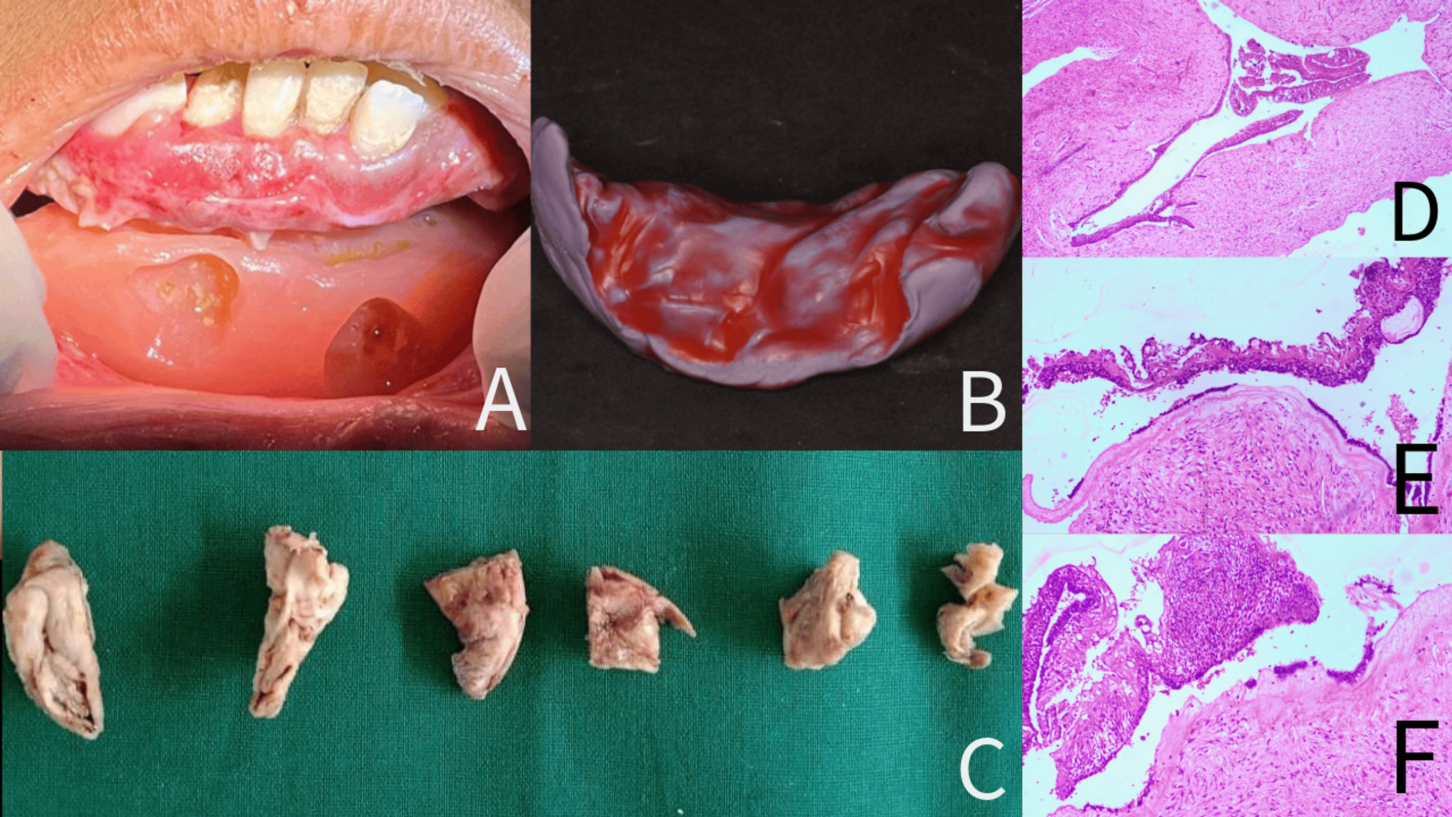
Obturator, gross specimen, and histopathological pictures of the case A) The obturator fitting into the area of the defect from postoperative day one. B) Fabrication of the obturator. C) Gross specimen that was sent for histopathological examination. D) Photomicrograph showing cystic epithelium separated from the connective tissue (4X, H&E). E) Photomicrograph showing subepithelial hyalinization and prominent palisaded basal cells with the suprabasal layer showing stellate reticulum-like cells (4X, H&E). F) Cystic epithelium also shows tall columnar basal cells and suprabasal cells showing a stellate reticulum-like appearance and subepithelial hyalinization (10X, H&E).

**Figure 3:**
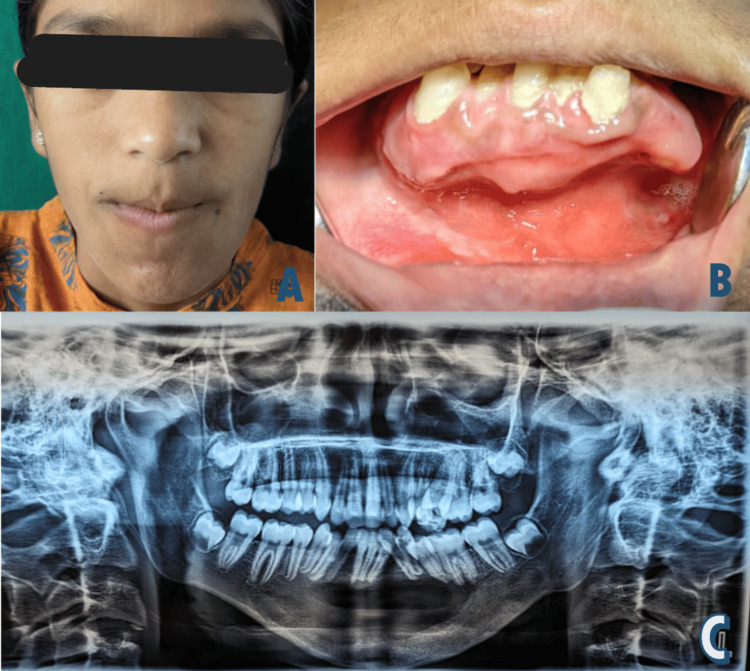
Postoperative clinical and radiographical pictures of the case A) Clinical picture of the one-year follow-up. B) Intraoral picture showing healing granulation tissue on one-year follow-up. C) Orthopantamogram at the one-year follow-up, with good healing and no signs of recurrence or infection. Written informed consent to publish the patient's image in an open-source journal has been obtained from the patient's legal guardian.

The procedure was performed under general anesthesia with nasal intubation. A vestibular incision was made from the 46 to 38 teeth region, and periosteal reflection was carefully done to expose the thin underlying bone. A small bony window was created on the labial plate, which was very thin, and the fluid in the cavity was suctioned. A bony fenestration was created in the buccal side to reveal the cystic epithelium, contents of the cyst were aspirated, and the cystic epithelium was enucleated. The intact lingual and basal bone were preserved to act as a scaffold for future osteointegration. The excised sample was sent for histopathological evaluation. Carnoy’s solution was applied for five minutes to reduce recurrence risk, followed by thorough irrigation with saline and a 5% povidone-iodine solution. An intraoperative alginate impression of the defect was taken to prepare an obturator. Following this, the cystic cavity was packed with a 5% povidone-iodine-soaked gauze and interrupted black silk sutures were used to close the defect. A nasogastric feeding tube was placed to prevent postoperative infection and removed after one week. Sutures were removed the day after the surgery, and the obturator was provided to the patient (Figures 2A, 2B). Daily irrigation was performed for the first two weeks, with follow-up appointments scheduled every two weeks. The obturator was gradually trimmed for easier insertion. One year follow-up of the patient showed adequate healing with bone formation (Figures 3A-3C).

Histopathology

Gross examination of the specimen included multiple hard and soft tissues, brownish-white in color, soft to firm in consistency with respect to soft tissue, whereas hard tissue appeared to be cortical plates and was hard in consistency. All the specimens were fixed in 10% neutral buffered formalin. The hard tissue was taken for decalcification, whereas the soft tissue was taken for routine processing and staining (Figure 1C).

Histopathologically, the specimen showed a cystic lining surrounded by a cystic capsule. The cystic lining was separated from the fibrous wall and the lining was a thin odontogenic epithelium with multiple extensions into connective tissue. The cystic epithelium was para-keratinized stratified squamous epithelium with surface corrugation and was separated from the connective tissue. Cystic epithelium also showed tall columnar basal cells exhibiting reversal of polarity, hyperchromatic nuclei with palisading arrangement, and sub-nuclear vacuolization suggestive of ameloblast cells. The superficial layer showed cells resembling stellate reticulum. Subepithelial hyalinization was also seen (Figures 3D-3F). Few intramural nodules in the connective tissue capsule were noted, resembling ameloblastic islands. Connective tissue shows diffused infiltration of chronic inflammatory cells, and blood vessels were also seen. Based on the histopathology, a final diagnosis of OKC with an intramural variant of unicystic ameloblastoma was made.

## Discussion

OKC was first described by Phillipsen in 1956 and is a distinctive cystic lesion of the maxilla and mandible. OKCs are aggressive lesions; therefore, a better understanding of the clinical characteristics and genetic and molecular factors is required. In one of the reported cases, the OKC extended from the mandible to the base of the skull, exhibiting behavior similar to a lower-grade squamous cell carcinoma.

OKCs are typically found in the mandible, with most cases occurring in the angle and ramus regions. The present case was located in the mandible extending from 38 to 46. OKCs are known to be associated with complications and are likely to transform into squamous cell carcinoma, basal cell carcinoma, ameloblastoma, or a component of the nevoid basal cell carcinoma (Gorlin's) syndrome.

The present case also showed one such complication wherein OKC showed transformation into unicystic ameloblastoma. This transformation underscores the complexity of diagnosing unicystic ameloblastomas, which can mimic other lesions, and emphasizes the need for a thorough and multidisciplinary diagnostic approach to distinguish it from other cysts, tumors, or bone dysplasias [8].

Although OKCs typically do not cause cortical expansion, our case presented with cortical plate expansion, suggesting aggressive behavior. Both OKC and unicystic ameloblastoma show similarities in clinical, radiographic, and anatomical presentation, complicating the differential diagnosis [9]. Both tend to occur more frequently in the mandible, often appearing as unilocular lesions with or without a multilocular pattern on radiographs. Some diagnostic tools, such as contrast-enhanced CT scans, can provide clearer visualization of internal architecture and highlight intraluminal components resulting from tumor wall growth. Clinically and radiographically, both types of ameloblastomas - the unicystic ameloblastoma and OKC - are indistinguishable due to their similar location of occurrence and the age of patients.

The literature has documented cases where two distinct tumors appear simultaneously in a patient's jaw. The rare simultaneous occurrence of ameloblastoma and OKC as distinct lesions has been documented. Previous reports have documented the co-occurrence of ameloblastomas with ortho-keratinized odontogenic cysts and glandular odontogenic cysts. Fregnani et al. reported synchronous ameloblastomas and ortho-keratinized odontogenic cysts, whereas Hisatomi et al. described a glandular odontogenic cyst associated with ameloblastoma. However, to our knowledge, there has been one case report of the transformation of an OKC into a unicystic ameloblastoma at the anterior mandible region involving the anterior teeth, but our case showed more aggressive behavior and showed extension of the lesion from 36 to 48 [5,10]. In this report, the primary challenge was to differentiate between unicystic ameloblastomas and OKC, as well as epithelial and mesenchymal tumors commonly affecting the jaws. Factors such as the age of the patient, the appearance of a multilocular pattern, and the involvement of the mandibular midline, unusual for many odontogenic tumors, were considered in the diagnostic process.

Genetic mutations and molecular changes may play a key role in the transition from a cystic lesion to a more aggressive tumor. Additionally, changes in the local microenvironment, such as chronic inflammation and tissue remodeling, might create conditions favorable for neoplastic transformation, particularly in recurrent OKCs [11]. Another possibility is the involvement of pluripotent stem cells within the cystic lining, which could differentiate into ameloblastic cells under certain conditions, leading to the development of unicystic ameloblastoma. Each of these hypotheses underscores the need for further research to fully understand the underlying mechanisms of this transformation. Combined odontogenic lesions of the jaws are complex and can be easily misdiagnosed. Accurate identification of the lesion is essential before establishing an appropriate treatment plan. Histopathological and immunohistochemical examinations of biopsy specimens are crucial for both accurate diagnosis and prognosis [12]. According to Jeyaraj P et al., immunohistochemistry is a valuable tool, particularly with tumor markers like "calretinin," which is specific to ameloblastomatous cells. This marker serves as a diagnostic tool to distinguish between odontogenic tumors and cysts [13]. However, in the present case, we did not do any immunohistochemical analysis since histopathology was pathognomonic of unicystic ameloblastoma and OKC.

The treatment of OKC ranges from enucleation and curettage to segmental resection. Philipsen and Reichart classified ameloblastomas into luminal and intraluminal types, which are treated conservatively, and intramural types, which require radical resection. Nick et al. found that Carnoy’s solution is beneficial for routine cases if follow-up is likely while decompression or marsupialization followed by enucleation is effective for large cysts with low recurrence rates. For patients unlikely to adhere to follow-up, resection is recommended [11]. However, the final decision of treatment depends on key factors like the patient’s age, cyst size, location, behavior, appearance on radiographs, presence of perforation, or soft tissue involvement, commitment to anatomical structures, and possible complications. Since the present case was seen in a young female and incisional biopsy diagnosis, we had opted to perform enucleation and curettage with adjuvant therapy of Carnoy's solution. Considering the patient's age and the desire to avoid more radical measures such as segmental resection, treatment was carried out with the preservation of teeth. An obturator was given from postoperative day one to cover the defect area.

Long-term follow-up is recommended due to the potential for late recurrence of OKCs [14,15]. It is crucial, particularly within the first year when most recurrences occur, and subsequent follow-ups every two years for at least 25 years to monitor for any late recurrences. We are currently following up with the patient. This case enlightens the multidisciplinary approach involving oral surgeons, pathologists, and radiologists is essential for effective diagnosis and management.

Prasath Jeyaraman et al. validated differentially upregulated expression of matrix proteins FN1, COL I, and IGF-1 in ameloblastoma relative to OKC because all three genes and related proteins were more highly expressed throughout the tumor stroma in ameloblastoma. They identified multiple differentially expressed genes between ameloblastoma and OKC. Jira Kitisubkanchana et al. suggested that a radiolucent lesion showing a smooth border, unilocular shape, no adjacent tooth displacement, and no root resorption, with mild or no bone expansion, is likely to be an OKC rather than an ameloblastoma. These radiographic findings might be helpful for differential diagnosis between them. Pereira et al. concluded that high expression of SOX2 in OKC indicates the presence of stem cells with significant self-renewal and proliferative properties, potentially signifying neoplastic behavior. In contrast, weak or absent expression of SOX2 in ameloblastoma suggests different molecular pathways involved in its neoplastic behavior [16-18].

This case highlights the critical role of histopathology in confirming the final diagnosis, especially when radiographic and clinical findings suggest a benign cystic lesion. The identification of ameloblastic features within the lesion transformed the diagnosis from OKC to unicystic ameloblastoma, emphasizing the potential for diagnostic overlap and the need for meticulous evaluation.

This case report discusses a young patient presenting with extraoral facial asymmetry and a multilocular radiolucent lesion in the anterior-posterior mandible, displacing roots of mandibular teeth (38-46) with cortical plate expansion. While preoperative imaging suggested an odontogenic keratocyst (OKC), histopathological analysis confirmed a unicystic ameloblastoma, necessitating more vigilant management due to its neoplastic potential. While OKC was the most likely diagnosis preoperatively, histopathological analysis revealed the lesion’s transition into a unicystic ameloblastoma, a less likely but significant diagnosis requiring more vigilant follow-up and management to address its neoplastic potential. The treatment prioritized tooth and bone preservation along with complete cyst removal. At the one-year follow-up, the surgical site showed excellent healing, highlighting a favorable prognosis. Regular postoperative monitoring and histopathological confirmation remain critical, with alternative treatments like marsupialization considered to minimize surgical intervention for younger patients.

Hence, this case is remarkable due to its unique presentation in terms of age, site of occurrence, and the clinical, radiological, and histopathological characteristics associated with the transformation of an OKC into unicystic ameloblastoma, a rare phenomenon. Documenting such a case provides an opportunity to enhance our understanding of this uncommon transformation, improve diagnostic accuracy, and refine treatment strategies. Additionally, it portrays the importance of ongoing research and collaboration among specialists to address complex odontogenic lesions effectively.

## Conclusions

The present case report highlights the importance of thorough investigations of odontogenic cysts and tumors occurring in the jaw bones. This case presented both clinically and histopathologically as aggressive lesions showing bone expansion and occurrence of OKC and unicystic ameloblastoma, which are known for recurrence. One year follow-up of the patient showed adequate healing with no recurrence, suggesting the treatment plan had done justice to both the patient and the surgeon. However, the patient is kept on long-term follow-up.
